# Immunosuppression abrogates resistance of young rabbits to Rabbit Haemorrhagic Disease (RHD)

**DOI:** 10.1186/1297-9716-45-14

**Published:** 2014-02-04

**Authors:** Raquel M Marques, Luzia Teixeira, Artur P Águas, Joana C Ribeiro, António Costa-e-Silva, Paula G Ferreira

**Affiliations:** 1 Department of Anatomy, ICBAS (Abel Salazar Institute for Biomedical Science) and UMIB (Unit for Multidisciplinary Biomedical Research), University of Porto, Rua de Jorge Viterbo Ferreira n.° 228, 4050-313 Porto, Portugal

## Abstract

Rabbit Haemorrhagic Disease (RHD) is caused by a calicivirus (RHDV) that kills 90% of infected adult European rabbits within 3 days. Remarkably, young rabbits are resistant to RHD. We induced immunosuppression in young rabbits by treatment with methylprednisolone acetate (MPA) and challenged the animals with RHDV by intramuscular injection. All of these young rabbits died within 3 days of infection due to fulminant hepatitis, presenting a large number of RHDV-positive dead or apoptotic hepatocytes, and a significant seric increase in cytokines, features that are similar to those of naïve adult rabbits infected by RHDV. We conclude that MPA-induced immunosuppression abrogates the resistance of young rabbits to RHD, indicating that there are differences in the innate immune system between young and adult rabbits that contribute to their distinct resistance/susceptibility to RHDV infection.

## Introduction, methods and results

Rabbits of the species *Oryctolagus cuniculus* are the natural hosts of the Rabbit Haemorrhagic Disease Virus (RHDV). This virus targets the liver of rabbits and causes the death of millions of wild and domestic adult rabbits worldwide [1-3]. Usually, adult rabbits die within 3 days of RHDV infection as a result of fulminant hepatitis, showing no symptoms of the disease until a few hours before death. Interestingly, young rabbits (less than 4 weeks-old) are resistant to Rabbit Haemorrhagic Disease (RHD), developing only a sub-clinical disease to the viral infection [3-6]. A central issue in the pathogenesis of RHD is to understand this age-related resistance to a viral infection that is fatal in adult animals. Pertinent to this issue, Ruvoën-Clouet et al. [7] reported that RHDV is capable of binding to histo-blood group antigens (HBGA) that are expressed on the mucosa of the upper respiratory and digestive tracts of adult rabbits, and they have postulated that the density of these attachment factors on mucosal cells is essential for adult rabbits to be susceptible to RHDV infection. In agreement with their view, they found only a weak binding of virus particles to the same mucosal tissues in young rabbits, thus indicating that low expression of HBGA could explain the resistance of young animals to RHD. Nevertheless, and despite this evidence, they recently reported that low expression of these facilitating factors of the infection, at the epithelial level, only confers partial protection against RHDV infection [3,8]. They also showed that hepatocytes, the main target of RHDV replication, do not express HBGA [7,8], which led them to suggest the existence of additional hepatic cellular receptor(s) for the virus [3]. Additionally, we demonstrated that young rabbits infected with RHDV by the intramuscular route develop the exact same mild liver disease that we had observed in young rabbits infected with RHDV by natural, oral and nasal routes [4,9-12]. This made us consider that resistance of young rabbits to RHD may depend on additional putative factors. A difference in innate immunity is one of these possible factors, since previous data have shown that young and adult rabbits develop different immune responses after RHDV infection [4-6,13-16]. Thus, we decided to study whether altering the immune physiology of young rabbits by methylprednisolone acetate (MPA)-induced immunosuppression would interfere with the resistance of young rabbits to RHDV infection.

New Zealand white rabbits (*Oryctolagus cuniculus*) were used when the animals were 4 weeks old. The rabbits were kept under standard conditions of housing with unrestricted access to food and water; this was done according to the European Union Directive no. 2010/63/CE and was approved by the appropriate local ethic committee (“Comité de Ética do ICBAS/UP”). The inoculum of RHDV (Ast/89 strain) was obtained as previously described by Teixeira et al. [17] and had a 2^10^ titre in haemagglutination units (HAU). Fourteen young rabbits (24 days) were immunosuppressed with an intramuscular injection of a suspension of MPA a long-acting corticosteroid (Depo-Medrol**®**; 20 mg/kg). All animals were also injected subcutaneously with a broad spectrum antibiotic, enrofloxacine (Alsir**®** 2.5%; 10 mg/kg), in order to prevent secondary bacterial infections. One week later, 9 of the 14 immunosuppressed rabbits were challenged with 100 μL of RHDV injected intramuscularly. The other 5 young rabbits were used as controls of the immunosuppression, i.e. they were euthanized and their thymuses were collected and weighed in relation with the body weight of the rabbits [18]. Another group of 15 young rabbits were used as the control of the infection or of the experiment. Ten animals were inoculated, injected intramuscularly, with 100 μL of RHDV, and the other 5 were inoculated, injected intramuscularly, with 100 μL of PBS, at the same time of the immunosuppressed rabbits. Five of the infected rabbits also received the antibiotic, according to the same scheme of the immunosuppressed rabbits. All the animals were observed regularly, and clinical manifestations of RHD were monitored. After the animal’s death, by disease or euthanasia (as described by Marques et al. [16]), the presence of macroscopic lesions compatible with RHD was investigated. Fragments of the thymus, spleen and liver were collected for histopathological and TUNEL analysis as described below. Blood samples were obtained from the vena cava caudal in order to quantify the seric level of cytokines (TNF-α, IL-6 and IL-10) by ELISA (ELISA Kit, Uscn Life Science Inc., Wuhan, People’s Republic of China) in 3 of the experimental groups: PBS control rabbits, immunosuppressed rabbits and immunosuppressed, RHDV-infected young rabbits. Liver samples of immunosuppressed, RHDV-infected young rabbits and infected young rabbits were also collected to quantify the presence of RHDV antigen by ELISA (the viral titer corresponding to the threshold value of dilution at which the sample is considered positive to RHDV, i.e., when its value is superior to the calculated cut-off (0.2 × OD_405nm_ positive control sample; INGEZIM RHDV 17.RHD.K2 kit, Ingenasa, Madrid, Spain). Other liver samples were fixed by 10% buffered formalin, dehydrated through graded alcohols and xylene and embedded in paraffin wax by standard methods. These were used for light microscopy studies that included immunocytochemistry for RHDV antigen (protocol described by Marques et al. [16]) and TUNEL assay for apoptosis detection (protocol described according to the manufacturer’s instructions (TdT in situ - DAB In Situ Apoptosis Detection Kit, R&D Systems Europe, Ltd., Abingdon, United Kingdom)). Samples of the spleen and thymus were also fixed with 10% buffered formalin to be used for apoptosis detection by the TUNEL assay. Statistical comparison of data from all groups of rabbits was performed using an unpaired t-test calculated with GraphPad Prism, version 5.0a, software. Differences were considered significant at *p* < 0.05.

**Figure 1:**
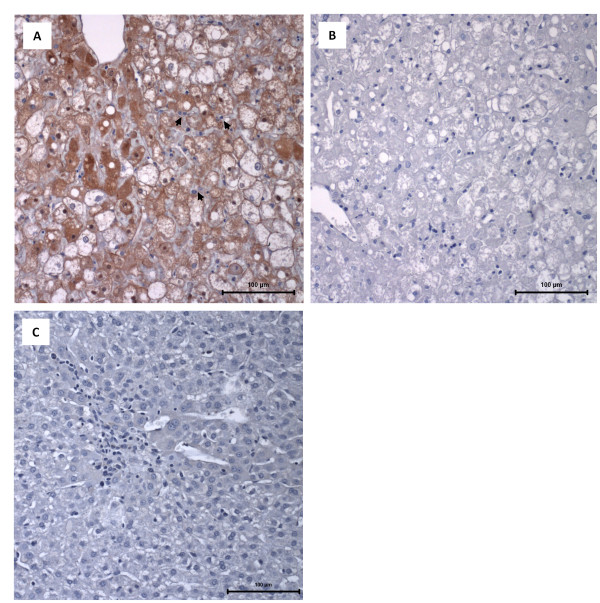
**Immunocytochemistry of RHDV antigen in the liver. A**: Light micrograph of a liver paraffin section of an immunosuppressed, RHDV-infected young rabbit that died with RHD showing widespread positive immunodetection of the major RHDV antigen (brown staining). Heterophils are observed surrounding the damaged hepatocytes and inside sinusoids (arrows). The tissue section was labelled with an anti-VP60 mouse antiserum; haematoxylin counterstain; bar = 100 μm. **B**: A liver section of an immunosuppressed, RHDV-infected young rabbit that died with RHD and that was labeled with control mouse antiserum; haematoxylin counterstain, bar = 100 μm. **C**: A liver paraffin section of an infected young rabbit that survived the infection and was euthanized 7 days later. Signs of hepatocellular regeneration are visible as expressed by enlarged and often binucleated cells (without the formation of organized hepatic cords); some tissue spaces are occupied by small basophilic cells, with an oval shape, that are suggestive of hepatocyte precursors. The tissue section was labeled with an anti-VP60 mouse antiserum; haematoxylin counterstain, bar = 100 μm.

**Figure 2:**
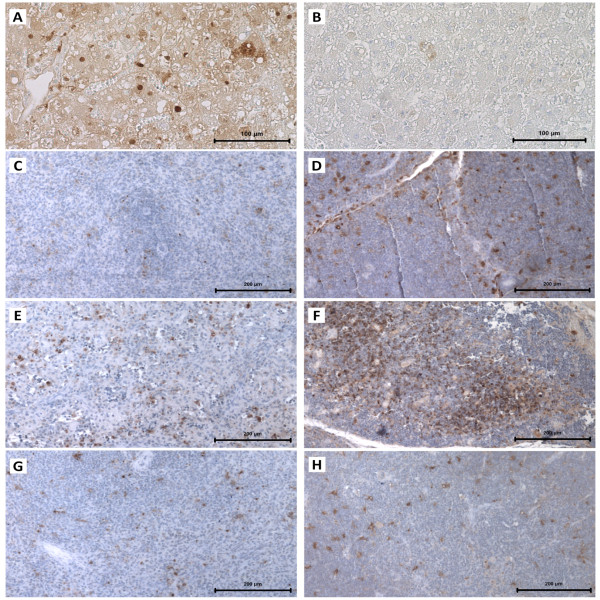
**Immunocytochemistry of apoptosis in the liver, spleen and thymus.** Light micrographs of the liver, spleen and thymus paraffin sections where the immunodetection of cellular apoptosis was obtained by TUNEL; counterstaining with haematoxylin. In liver sections, bar = 100 μm; in spleen and thymus sections, bar = 200 μm. **A**: A liver paraffin section of an immunosuppressed, RHDV-infected young rabbit that died due to RHD. **B**: A liver paraffin section of an immunosuppressed, RHDV-infected young rabbit that died from RHD; control of the TUNEL assay. **C**: A spleen paraffin section of an immunosuppressed young rabbit euthanized 7 days after immunosuppression. **D**: A thymus paraffin section of an immunosuppressed young rabbit euthanized 7 days after immunosuppression. **E**: A spleen paraffin section of an immunosuppressed, RHDV-infected young rabbit that died from RHD. **F**: A thymus paraffin section of an immunosuppressed, RHDV-infected young rabbit that died from RHD. **G**: A spleen paraffin section of an infected young rabbit euthanized 7 days after RHDV infection. **H**: A thymus paraffin section of an infected young rabbit euthanized 7 days after RHDV infection.

**Figure 3:**
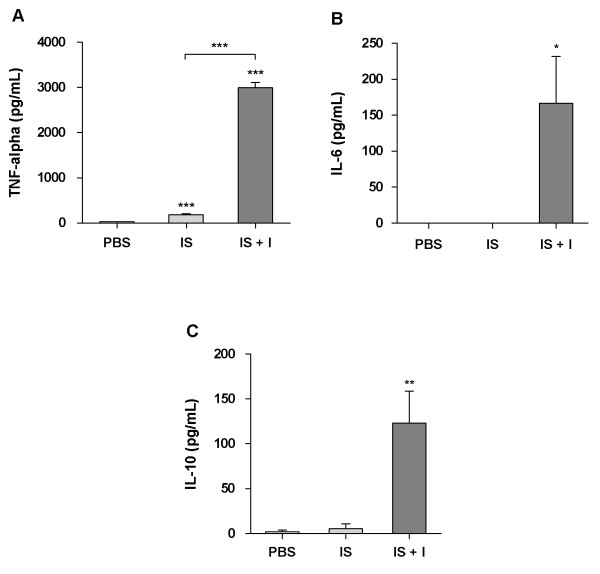
**Evaluation of serum cytokines *– *graphics. A**: Comparison of TNF-α levels in the serum of control (PBS) young rabbits (*n* = 5), immunosuppressed (IS) young rabbits (*n* = 5), and immunosuppressed RHDV-infected (IS + I) young rabbits (*n* = 9) that died from RHD; unpaired t test, (***p < 0.0001); the results are shown as a mean ± SEM). **B**: Comparison of IL-6 levels in the serum of control (PBS) young rabbits (*n* = 5), immunosuppressed (IS) young rabbits (*n* = 5) and immunosuppressed RHDV-infected (IS + I) young rabbits (n = 9) that died from RHD; unpaired t test, (*p < 0.05); the results are shown as a mean ± SEM). **C:** Comparison of IL-10 levels in the serum of control (PBS) young rabbits (*n* = 5), immunosuppressed (IS) young rabbits (*n* = 5), and immunosuppressed RHDV-infected (IS + I) young rabbits (*n* = 9) that died from RHD; unpaired t test, (**p < 0.01); the results are shown as a mean ± SEM).

All immunosuppressed, RHDV-infected young rabbits died within 24–72 h of RHDV infection (24–30 h *n* = 2; 36–48 h *n* = 6; 60–72 h *n* = 1) depicting the hyperacute form of RHD, i.e., they died suddenly, often without conspicuous signs of disease, showing prostration, lethargy and anorexia. As expected, all non-immunosuppressed infected young rabbits showed no clinical symptoms of disease after being infected with RHDV; they were euthanized 1 week after the viral inoculation. Immunosuppressed rabbits (infected (IS + I) or not (IS)) showed atrophic thymuses, indicating that immunosuppression had been achieved; this atrophy was confirmed by the ratio thymus (g)/body weight (g) since we found that these ratios were significantly lower in immunosuppressed groups (IS group: 0.00048 ± 0.000049; IS + I group: 0.00054 ± 0.000050; mean ± standard error of the mean (SEM); *p* < 0.0001) than in control rabbits (PBS group: 0.0024 ± 0.00024; mean ± SEM). The immunosuppressed, RHDV-infected young rabbits showed the typical lesions of RHD, such as enlarged and pale livers with enhanced lobular patterns, congestion and haemorrhagic lesions of several viscera, and bloody foam in the trachea. A significant difference was seen in the spleen volume, with splenomegaly in immunosuppressed, RHDV-infected rabbits in contrast with spleen atrophy in immunosuppressed animals (ratio - spleen (g)/body weight (g); *p* < 0.05). Young rabbits that were infected with RHDV did not present any macroscopic lesions suggestive of RHD, 7 days after the viral inoculation, when they were euthanized. Widespread liver cell vacuolization was seen in immunosuppressed, RHDV-infected young rabbits; these hepatocytes often showed pyknotic nuclei and karyorrhexis, classic features of cell death (Figure 1A). The TUNEL assay confirmed extensive cell death of hepatocytes and this was illustrated by brown-nuclear staining (early apoptotic cells) or brown-cytoplasmic staining (late apoptotic or necrotic cells) (Figure 2A). Scattered inflammatory cells (predominantly heterophils) were seen in the liver of the animals. In contrast, hepatic tissue of the immunosuppressed rabbits did not reveal any signs of hepatocellular degeneration suggestive of apoptosis. In RHDV-infected young rabbits, at day 7^th^ after viral inoculation, there was evidence of hepatic regeneration as expressed by rebuilding of focal hepatic lesions that had been produced by the infection (Figure 1C). All immunosuppressed, RHDV-infected young rabbits presented an extensive brown-staining of the liver for the RHDV antigen (Figure 1A). In contrast, RHDV-infected young rabbits, euthanized 7 days after infection, exhibited, in general, no RHDV-positive staining (Figure 1C). ELISA revealed that all immunosuppressed, RHDV-infected young rabbits presented elevated viral titers in the liver (240.0 ± 99.06; mean ± SEM), similarly to those found in RHDV-infected adult rabbits that died spontaneously with RHD (170.7 ± 42.67; mean ± SEM; *n* = 3; data obtained from previous experiments). In contrast, the virus was not detected in the livers of young rabbits that had been submitted to the RHDV infection. The TUNEL assay confirmed marked apoptosis in the thymus of immunosuppressed, RHDV-infected rabbits and this was less extensive in the spleen (Figures 2E-F); these tissue changes were not observed in infected young rabbits not submitted to immunosuppression (Figures 2G-H). As expected, significant apoptosis of thymus lymphocytes was observed in all immunosuppressed animals (Figure 2D). The values of the proinflammatory cytokines, TNF-α, IL-6, and the anti-inflammatory cytokine, IL-10, were determined in the sera of PBS control rabbits, immunosuppressed rabbits and immunosuppressed, RHDV-infected young rabbits. All cytokines had a marked increase in the serum of immunosuppressed, RHDV-infected young rabbits that died with RHD, TNF-α in particular (Figure 3).

## Discussion

RHDV infection kills most adult rabbits, whereas young rabbits show no disease [4,5,12]. Our previous reports on the pathogenesis of RHD [5,6,14-16] indicated that young and adult rabbits may differ in innate immune response, since the adult animals usually die in less than 3 days (a too early timing for the host to mount a specific immune response to the infectious agent). To investigate a putative role of innate immunity in the resistance of young rabbits, we decided to investigate whether immunosuppression would change the progress of calicivirus infection in these animals. For that, we studied rabbits treated with MPA, a corticosteroid of long-duration, with pleiotropic actions including immunosuppression due to inhibition of the inflammatory response and depletion of both T and B cells [19,20].

We found that young rabbits under MPA-immunosuppression died from RHDV infection within 3 days, just as naïve adult rabbits do after RHDV inoculation. The animals died from fulminant hepatitis that was associated with widespread apoptosis and disseminated intravascular coagulation (DIC). The inflammatory infiltrates in the liver of the immunosuppressed, RHDV-infected young rabbits were mainly constituted by heterophils, contrary to what would be expected in infected young rabbits, i.e., where liver infiltrates contain macrophages and lymphocytes [3,4,6]. These features mimic the typical pathology of RHD in adult rabbits after being infected by RHDV [12,14,21]. Also, similarly to infected adult rabbits, the immunosuppressed, RHDV-infected young rabbits showed a marked dissemination of RHDV throughout the liver. The viral titers registered in the liver of immunosuppressed, RHDV-infected young rabbits were also similar to those found in infected adult rabbits. These data show that young rabbits that are treated with MPA develop the full blown RHD seen in adult animals after RHDV infection. This change of young rabbits from resistance to susceptibility to the calicivirus is likely to result from impairment of their innate immunity, i.e., the MPA treatment could, for example, be interfering with the process of antigen presentation, with the phagocytic activity, or with the effector roles of activated NK cells, NK T cells or possibly CD8^+^ T cells [22-25]. Our results weaken the hypothesis that resistance of young rabbits to RHD derives from a different expression of the viral hepatic cellular receptor(s), since the MPA treatment of the young rabbits made the RHDV capable of infecting the hepatocytes, as observed in adult rabbits.

We also report here a marked increase in proinflammatory and anti-inflammatory cytokines (TNF-α, IL-6 and IL-10) in the serum of the immunosuppressed, RHDV-infected young rabbits. This enhancement in cytokines is similar to what occurs in the blood of infected adult rabbits a few hours prior to death. Others have previously shown that RHDV infection of adult rabbits is related to a strong increase of mRNA levels of pro-inflammatory cytokines in the liver, target organ of viral replication [26,27]. The histopathological signs of fulminant hepatitis observed in the immunosuppressed, RHDV-infected young rabbits, similarly to what happens in adult rabbits having died from RHD, involved numerous apoptotic liver cells that are due to the cytopathic effect of viral replication and the exacerbated inflammatory response caused by the deregulated expression of cytokines detected in these animals, TNF-α in particular, since it is a well-known cell death mediator [26,27]. A similar phenomenon is seen in B hepatitis, where secretion of TNF-α by macrophages and infected hepatocytes contributes to liver cell death [28,29]. In contrast, young rabbits that are infected with RHDV present an early and only mild increase of proinflammatory cytokines [16]. This corroborates the idea that RHDV infection in young rabbits is associated with an early and adequate inflammatory immune response that may be vital in strongly limiting viral replication, and consequently allowing the more than 3 days that are required for the rabbits to develop a specific immune response against RHDV, similarly to what is observed in the few survivors of Ebola virus infection [10,30].

In conclusion, we demonstrate that the innate immune response of young rabbits is abrogated by the MPA treatment, changing young rabbits into hosts that become as susceptible to fatal calicivirus infection as naïve adult rabbits are. Thus we propose that the resistance of young rabbits to RHDV-infection is mediated by their particular innate immunity that is different from that of adult rabbits. Moreover, this discovery opens the way for a better understanding of the rationale of resistance of young rabbits to RHD.

## Competing interests

The authors declare they have no competing interests.

## Authors’ contributions

RMM designed the study, performed the animal experiments, wrote the manuscript and performed the statistical analysis. LT participated in the animal experiments, helped in the immunohistochemistry analysis and drafting the manuscript. JCR performed the ELISA and TUNEL assays. ACS participated in the animal experiments. APA participated in the study design, interpretation of the results and helped draft the manuscript. PGF contributed to the elaboration of the study design, interpretation of the results and helped draft the manuscript. All authors read and approved the final manuscript.
